# Enzymatic Bioweathering and Metal Mobilization From Black Slate by the Basidiomycete *Schizophyllum commune*

**DOI:** 10.3389/fmicb.2018.02545

**Published:** 2018-10-24

**Authors:** Julia Kirtzel, Soumya Madhavan, Natalie Wielsch, Alexander Blinne, Yvonne Hupfer, Jörg Linde, Katrin Krause, Aleš Svatoš, Erika Kothe

**Affiliations:** ^1^Microbial Communication, Institute of Microbiology, Friedrich Schiller University, Jena, Germany; ^2^Research Group Mass Spectrometry/Proteomics, Max Planck Institute for Chemical Ecology, Jena, Germany; ^3^Helmholtz Institute Jena, Jena, Germany; ^4^Hans Knöll Institute, Jena, Germany

**Keywords:** *Schizophyllum commune*, bioweathering, proteome, laccases, multicopper oxidases, rock, black slate

## Abstract

*Schizophyllum commune* is a filamentous basidiomycete causing white-rot in many wood species with the help of a broad range of enzymes including multicopper oxidases such as laccases and laccase-like oxidases. Since these enzymes exhibit a broad substrate range, their ability to oxidatively degrade black slate was investigated. Both haploid monokaryotic, and mated dikaryotic strains were able to grow on black slate rich in organic carbon as sole carbon source. On defined media, only the monokaryon showed growth promotion by addition of slate. At the same time, metals were released from the slate and, after reaching a threshold concentration, inhibited further growth of the fungus. The proteome during decomposition of the black slate showed induction of proteins potentially involved in rock degradation and stress resistance, and the gene for laccase-like oxidase *mco2* was up-regulated. Specifically in the dikaryon, the laccase gene *lcc1* was induced, while *lcc2* as well as *mco1, mco3*, and *mco4* expression levels remained similar. Spectrophotometric analysis revealed that both life forms were able to degrade the rock and produce smaller particles.

## Introduction

In lignin degradation performed by white-rot fungi like *Schizophyllum commune*, peroxidases and multicopper oxidases are involved. The lignin degrading enzymes (FOLymes, Levasseur et al., 2008) can be identified from fungal genomes, and 16 FOLymes including two laccases and four other multicopper oxidases were identified for *S. commune* (Ohm et al., 2010). Compared to other white-rot fungi, *S. commune* lacks genes encoding peroxidases and thus features a slim FOLyme repertoire (Ohm et al., 2010).

Multicopper oxidases, including true laccases, show a wide range of substrate utilization and thus are involved in various degradative and biosynthesis processes. With two laccases (*lcc1* and *lcc2*) and four laccase-like multicopper oxidases (*mco1, mco2, mco3*, and *mco4*), each containing copper binding domains with the typical four laccase signature sequences (Madhavan et al., 2014), *S. commune* seems well equipped for acting on an array of organic carbon fractions occurring in nature including wood, but also bitumen or kerogen. Kerogen is the altered form of lignin presentin sedimentary rocks like shales and slates. Indeed, previous studies have confirmed the ability of *S. commune* to degrade black slate material resulting in a release of heavy metals and carbon as dissolved organic matter (Wengel et al., 2006; Kirtzel et al., 2017).

Black slates are low-grade metamorphic rocks rich in sulfides, heavy metals and high amounts of organic matter altered into kerogen. Black slates and shales are important to nature as they are a prominent reservoir of organic carbon and experience several changes when they are exposed to oxygen. Weathering of such organic matter is essential for the geochemical cycles of carbon and oxygen, and fungal involvement in this process may lead to fast weathering of the rock material (Fischer et al., 2007). Due to their metal contents, black slates have been mined for aluminum, copper or vitriol production, but also for production of uranium. In Thuringia, Germany, the former vitriol mines at Saalfeld and Morassina and the former uranium mine near Ronneburg are examples for black slate mining activities. The high sulfide contents, especially at the former Ronneburg mining site, has led to acid mine drainage representing a major environmental challenge. The weathering leading to metal release and acidification through sulfide (pyrite) oxidation is often linked to bacterial activity (Mustin et al., 1992; Uroz et al., 2009; Konhauser et al., 2011).

Nevertheless, the activity of fungi can largely contribute to such bacterial activities as fungi are able to secrete organic and inorganic acids, and widen cracks in the rock and thus expose fresh surfaces to bacterial activity (Burford et al., 2003; Hoffland et al., 2004). This will be specifically important in dense rock material like black slates. However, in order to exert a force on the rock, in addition to directional growth, the fungus requires enzymatic degradation of the rock material to induce surface roughness which in turn enables hyphal attachment (Willmann and Fakoussa, 1997; Burford et al., 2003).

The structure of kerogen in black slates has been suggested to be similar to lignin components, explaining the observed lytic activities (Catcheside and Mallett, 1991; Hofrichter et al., 1997a,b). In accordance with this observation, fungi have been identified to depolymerize or transform coal (Hofrichter et al., 1997a,b; Petsch et al., 2001; Wengel et al., 2006). Here, we focus on the differential expression and enzymes active in the degradation of black slate.

The life cycle of basidiomycetes involves germination and haploid, monokaryotic mycelial growth from sexually formed basidiospores. If two compatible mates fuse, a dikaryon with two parental nuclei per cell is formed (Freihorst et al., 2016). This dikaryon can then form fruiting bodies under optimal environmental conditions, in which haploid basidiospores are produced, which makes it a good model to study sexual development (Raper and Miles, 1958; Kothe, 1997). *S. commune* is able to complete this life cycle on water agar with black slate as the only carbon and nutrient source within 2–3 weeks (Supplementary Figure S1).

The significance of fungi in bioweathering of rocks and minerals gained considerable attention during the last decades. However, only very few investigations deal with basidiomycetes and their enzymatic biodegradation. In the present study, we investigated the ability of *S. commune* to degrade black slate and use it as a carbon source. The effect of black slate on mono- and dikaryotic strains was examined, as well as the influence of the rock material on fungal growth and metabolism. Since the two life stages show different enzyme expression patterns, we performed proteome analyses to identify secreted proteins participating in black slate degradation. To the best of our knowledge, these results provide a first insight into possible rock decay mechanisms for *S. commune*. Detected multicopper oxidases were further investigated *via* an enzymatic assay and differential expression analysis during growth of both *S. commune* life stages on black slate.

## Materials and Methods

### Strains and Culture Conditions

Pre-cultures of *S. commune* monokaryon 4–39 and dikaryon 12–43 × 4–39 (Jena Microbial Resource Collection, Jena, Germany) were grown in liquid complex yeast extract medium (Raper and Hoffman, 1974). After 5 days, the mycelium was washed twice with water, homogenized and resuspended in 100 ml half diluted minimal medium (Raper and Hoffman, 1974). Of this, 7 ml were used to inoculate 200 ml half diluted minimal medium containing 3 g powdered black slate (0.5 g < 63 μm, 2.5 g 63 μm-1 mm fractions obtained by sieve analysis, collected from Schmiedefeld, a former alum mining area in Thuringia, Germany) and incubated at 28°C and 130 rpm. The specific surface area of black slate has been shown to vary between 10 and 20 m^2^/g (Fischer and Gaupp, 2004, 2005).

### Growth Rate

The effect of black slate on biomass production was determined for monokaryon and dikaryon in liquid medium using three biological replicates according to the above-mentioned culture conditions in presence and absence of black slate. Dry weight was determined over a period of 4 weeks and every black slate containing sample was normalized to the corresponding control containing no black slate. Fungal growth and the interaction with black slate were observed microscopically (Stemi 2000-C and LSM 780, Zeiss, Germany) 14 days after inoculation. At different points in time, mycelium and black slate were harvested by centrifugation at 11,000 rpm for 30 min and dried at 60°C. After subtracting the weight of black slate that had been added to the culture, the fungal biomass was calculated. To determine the effect of black slate for every replicate, the control biomass was subtracted from the appropriate biomass treated with black slate and the final change in growth was expressed in mg.

### Metal Release

Black slate containing liquid culture medium was analyzed to examine fungal-induced metal release from the rock material. Black slate containing medium inoculated with *S. commune* monokaryon was sampled after different points in time. Samples were filtered (0.22 μm, Rotilabo, Roth, Germany), acidified using HNO_3_ and analyzed by ICP-MS (X Series II, Thermo Fisher Scientific). Measurements were performed with three biological and three technical replicates using non-inoculated samples for control.

### Spectrophotometric Assay for Quantification of Black Slate Solubilization

Fungal solubilization of black slate was spectrophotometrically quantified by measuring organic substances such as (dissolved) humic substances released from black slate. Liquid cultures of monokaryon and dikaryon as well as controls without fungal biomass were incubated with and without powdered black slate and centrifuged for 5 min at 8000 rpm to allow settling of mycelium and large rock particles. The supernatant was transferred into cuvettes (Greiner Bio-One, Germany) and the absorbance was measured at 450 nm (Ultrospec 2100*pro*, Amersham Biosciences, Germany), the most suitable wavelength to determine dark-colored organic substances from black slate. Measurements were performed with three biological replicates and statistical analysis was achieved using paired, two-tailed *t*-test. Degraded black slate particles were microscopically examined (Axioplan 2 microscope, Zeiss, Germany) and their sizes were measured using SPOT advanced (Diagnostic Instruments, Germany).

### Protein Extraction

For preparation of protein extracts, liquid culture medium of four biological replicates was separated from fungal cultures and, when added, supplemented black slate 7 days after inoculation by centrifugation and 50 ml of the supernatant were concentrated to 1.5 ml (Amicon Ultra-15 Centrifugal Filter Units, 3 kDa, Merck Millipore, Germany). The supernatant was centrifuged at 12,000 ×*g* for 30 min to remove cell debris or insoluble compounds. Secreted proteins were precipitated by the addition of 10 % (w/v) trichloroacetic acid.

After centrifugation at 13,000 × g for 10 min at 4°C, the supernatant was discarded, the pellet washed twice in 90% acetone (-20°C) and subsequently dried at room temperature.

For shotgun analysis, the pellet was resuspended in 50 mM NH_4_HCO_3_ and prior trypsin (Roche) digestion the samples were reduced with 10 mM DTT at 56°C and alkylated with 55 mM iodoacetamide in the dark. A trypsin/protein ratio of 1:30 at 37°C was applied for approximately 12 h.

### NanoUPLC-MS/MS Analysis

The samples were acquired on UPLC M-class system (Waters Corporation) online coupled to a Synapt G2-si mass spectrometer equipped with a T-WAVE-IMS device (Waters Corporation). The peptides were first on-line preconcentrated and desalted using a UPLC M-Class Symmetry C18 trap column (100 Å, 180 μm × 20 mm, 5 μm particle size) at a flow rate of 15 μl/min (0.1% aqueous formic acid) and then eluted onto a ACQUITY UPLC HSS T3 analytical column (100 Å, 75 μm X 200, 1.8 μm particle size) at a flow rate of 350 nl/min with the following gradient: 1–9% B over 10 min, 9–19% B over 10 min, 19–32% B over 10 min, 32–48% B over 10 min, 48–58% over 5 min, 70–95% over 5 min, isocratic at 95% B for 4 min, and a return to 1% B over 1 min (phases A and B composed of 0.1% FA and 100% acetonitrile in 0.1% FA, respectively). The analytical column was re-equilibrated for 9 min prior to the next injection. The eluted peptides were transferred into the mass spectrometer operated in V-mode with a FWHM resolving power of at least 20000. All analyses were performed in a positive ESI mode. A 100 fmol/μl human Glu-Fibrinopeptide B in 0.1% formic acid/acetonitrile (1:1 v/v) was infused at a flow rate of 1 μl/min through the reference sprayer every 45 s to compensate for mass shifts in MS and MS/MS fragmentation mode.

During HDMS^E^ analysis, a wave height of 40 V was applied in IMS past of TriWave, and the traveling wave velocity was ramped from 1000 m/s to 600 m/s. Wave velocities in the trap and transfer cell were set to 311 m/s and 175 m/s and wave heights to 4 V and 4 V, respectively. For fragmentation, the collision energy was linearly ramped in the transfer region of TriWave from 20 to 45 V. The acquisition time in each mode was 0.5 s with a 0.05 s interscan delay. HDMS^E^ data were collected using MassLynx v4.1 software (Waters Corporation).

### Processing of LC-MS Data

Data analysis was performed using ProteinLynx Global Server (PLGS) version 2.5.2 (Waters Corporation). The thresholds for low/high energy scan ions and peptide intensity were set at 300, 30 and 750 counts, respectively. The processed data were searched against the *S. commune* protein database obtained from the Joint Genome Institute, and combined with a subdatabase containing common contaminants (human keratins and trypsin). The database searching was performed at a False Discovery Rate (FDR) of 2%, following searching parameters were applied for the minimum numbers of: fragments per peptide (1), peptides per protein (1), fragments per protein (3), and maximum number of missed tryptic cleavage sites (1). Searches were restricted to tryptic peptides with a fixed carbamidomethyl modification for Cys residues. For functional description of proteins, the Functional Catalog (FunCat) was used as classification system (Ruepp et al., 2004).

### Laccase Activity Assays

Extracellular laccase activity was determined from the culture fluid of liquid medium in presence and absence of black slate 7 days after inoculation (dpi, Sánchez et al., 2004), the point in time at which *S. commune* has been shown to release organic carbon from black slate (Wengel et al., 2006). Black slate powder was removed by centrifugation and extracellular enzymes were concentrated using membrane filters (Amicon Ultra-15 Centrifugal Filter Units, 10 kDa cutoff, Merck Millipore, Germany). Laccase activity was observed as increase in absorbance at 420 nm and expressed as units per liter (1 U = 1 μmol 2,2^′^-azino-bis (3-ethylbenzothiazoline-6-sulphonic acid), ABTS, oxidized per minute) with 1 mM ABTS in 100 mM sodium acetate buffer at pH 4.5 (Linke et al., 2005). Protein concentration was determined (Bradford, 1976). All tests were performed with three biological and three technical replicates and statistically analyzed using paired, two-tailed *t*-test.

### Expression Analyses

Approximately 100 mg of 7-day-old mycelium from monokaryotic or dikaryotic strains were harvested from the liquid black slate culture and washed properly. After grinding in liquid nitrogen, total RNA was isolated (Qiagen RNeasy Plant Mini Kit, Qiagen, Germany). For quantitative real-time PCR (qRT-PCR), the laccases *lcc1* (DOE JGI protein ID 2509814), *lcc2* (protein ID 1194451), laccase-like *mco1* (protein ID 2621035), *mco2* (protein ID 2634619), *mco3* (protein ID 2516955), *mco4* (protein ID 2483752) and reference genes *act1* (protein ID 1194206), *tef1* (protein ID 1037126), and *ubi* (protein ID 71656) were analyzed. Primers were used after Madhavan et al. (2014), and efficiencies were calculated from calibration curves (cDNA concentrations from 0.001 to 200 ng cDNA were used per 25 μl of PCR mixture). The qRT-PCR was performed using the Miniopticon Real Time PCR System (BioRad, Germany) and Maxima SYBR Green qPCR (Fisher Scientific, Germany) with initial denaturation at 94°C for 10 min, followed by 35 cycles of 94°C for 20s, 65°C for 20s (*lcc2* and *mco3*) or 60°C (for all other genes), and 72°C for 20 s. A melting curve analysis was performed for every run and each reaction was conducted with at least three biological and three technical replicates including negative controls, one without template and one without reverse transcription. PCR products were sequenced to verify the gene identity. The *C*_t_ values of target genes were normalized with respect to the reference genes, and calculated for relative and normalized fold change compared to treatments without black slate by the term 2^-ΔΔCT^ (Pfaffl, 2001; Vandesompele et al., 2002).

## Results

### Fungal Growth With Black Slate

**FIGURE 1:**
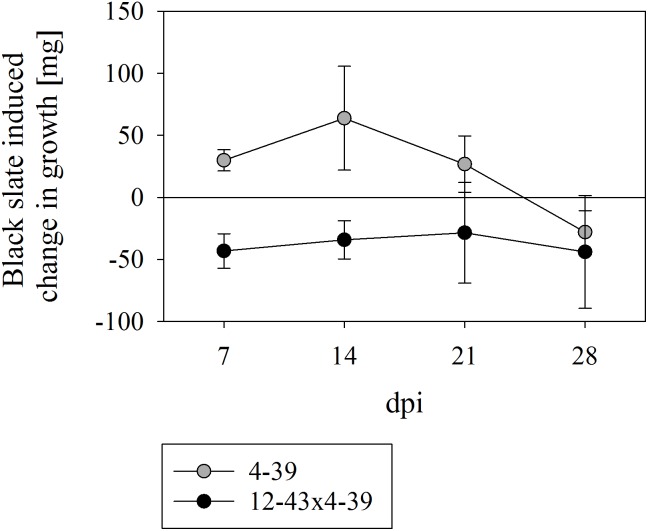
Growth rate of *Schizophyllum commune* strains 4–39 and 12–43 × 4–39 in presence of black slate normalized to the corresponding control without black slate supplement. Positive values indicate a black slate induced burst of growth and negative values reveal a black slate induced inhibition of growth; *dpi* days after inoculation. Error bars represent standard deviation of three biological replicates.

To test the impact of black slate on fungal growth, *S. commune* was grown with and without powdered rock supplemented to the minimal medium. Both mono- and dikaryons of *S. commune* were able to grow in liquid media with powdered black slate. The growth of the monokaryon was promoted by the addition of rock material during the first 21 days (with a peak in biomass production after 14 days, Figure 1). After that point, growth promotion of black slate decreased and a reverse effect, where black slate reduced the biomass production, was observed at 28 days. The dikaryon consistently exhibited a better growth in absence of the rock material, and no black slate induced change in growth was observed between 7 to 28 dpi.

**FIGURE 2:**
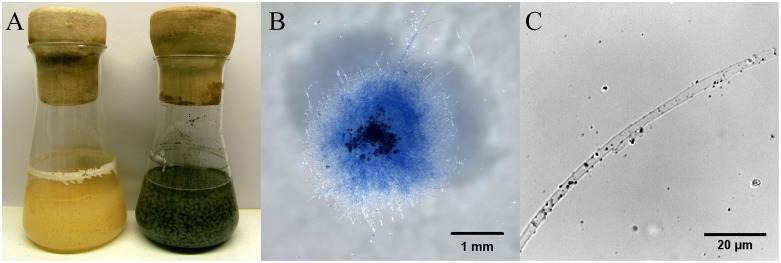
*S. commune* growing in half diluted minimal medium without and with fractionized black slate **(A)**, hyphal ball stained with Lactophenol Cotton Blue with inclusions of black slate **(B)**, and hypha of monokaryon 4–39 with attached small black slate particles **(C)** 14 dpi.

During growth in liquid medium, the mycelium of *S. commune* grew in mycelial balls which enclosed black slate particles (Figure 2). Most rock grains and mainly larger particles were located in the center of the grown mycelium, while smaller particles were found at lower incidence at the rim. A closer examination of exterior hyphae indicated the attachment and slight adherence of very small particles with an average size of 0.7 μm (Figure 2C).

### Metal Release

**FIGURE 3:**
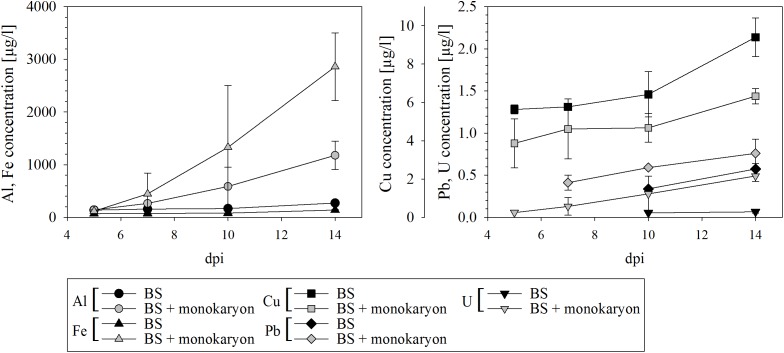
Metal release from black slate into culture medium of black slate containing medium (BS) serving as control and black slate containing medium with fungal inoculum (BS + monokaryon); *dpi* days after inoculation. Missing data points are below the detection limit. Error bars represent standard deviation of three biological replicates.

The ability of *S. commune* to release metals from black slate was confirmed with a significantly higher release of Al, Fe, and U and a slight release of Pb in presence of the fungus (Figure 3). A reverse effect, at which the monokaryon caused lower metal concentrations in the medium, was seen for Cu. Especially in presence of the fungus, increasing element concentrations were detected for all metals over time.

### Black Slate Degradation

**FIGURE 4:**
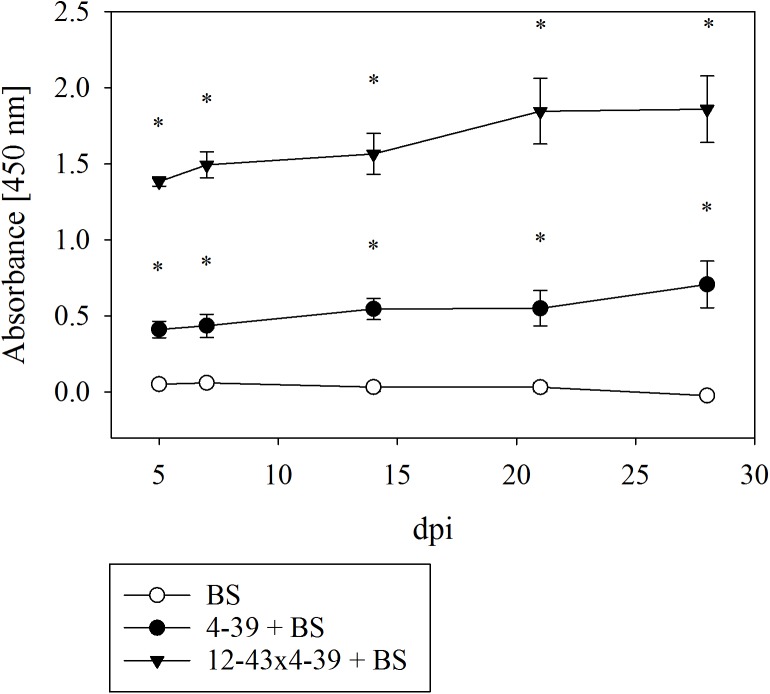
Spectrophotometric analysis for measuring the degradation of black slate (BS) by *S. commune*; *dpi* days after inoculation. Error bars represent standard deviation of three biological replicates; ^∗^*p* < 0.01, paired, two-tailed *t*-test.

The effect on rock degradation was verified by investigating the supernatants of liquid culture media with black slate. Bioweathering of black slate by *S. commune* cracked the rock into smaller pieces and caused the elevated release of dark-colored organic substances that may dissolve in the medium and can be spectrophotometrically measured. Both monokaryon and dikaryon caused a significantly higher absorbance at 450 nm due to organic substances and suspended black slate particles (Figure 4). The dikaryon *S. commune* 12–43 × 4–39 caused the highest turbidity of medium and the highest increase in absorbance 5–7 dpi; a less rapid but still escalating effect was seen up to 21 dpi, followed by a constant absorbance to 28 dpi. The monokaryon *S. commune* 4–39 showed a weaker, but similarly increasing absorbance pattern with a sharp rise in absorbance 5 dpi, and a slight increase in absorbance until 28 dpi.

Microscopic analyses demonstrated that both fungal strains caused a decomposition of black slate grains into smaller particles with an average size of 0.7 to 0.8 μm after 14 dpi. In presence of *S. commune*, these particles were present in the supernatant to a larger extent as compared to samples not inoculated with the fungus.

### Secretome Analysis

The secreted proteins of *S. commune* monokaryon 4–39 and dikaryon 12–43 × 4–39 were analyzed during cultivation with and without black slate. Both strains revealed the occurrence of 269 proteins, about 60% showed secretion motifs. For functional annotation, 157 proteins could be annotated. With 887 functions within 17 FunCat main categories, multiple function of most proteins was assigned.

**FIGURE 5:**
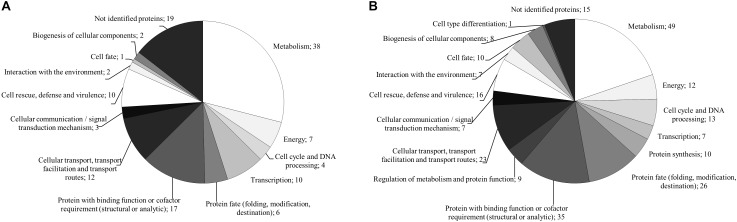
FunCat annotated functions of proteins exclusively produced in presence **(A)** and absence **(B)** of black slate of *S. commune* strain 4–39.

For the monokaryon *S. commune* 4–39, 38 of the 241 proteins were exclusively detected in presence of black slate (Supplementary Table S1). Their assigned functions mainly belonged to metabolism, to proteins with binding function or cofactor requirement, or to cellular transport, transport facilitation and transport routes (Figure 5A). Seventy proteins were detected in absence of black slate and exhibited functions in transport facilitation and transport routes, metabolism, protein fate, and protein with binding function or cofactor requirement (Figure 5B and Supplementary Table S2).

**FIGURE 6:**
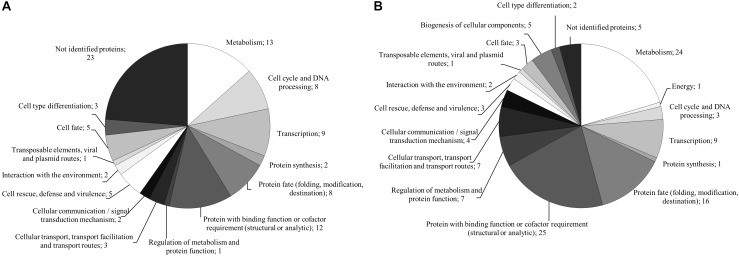
FunCat annotated functions of proteins exclusively produced in presence **(A)** and absence **(B)** of black slate of *S. commune* strain 12–43 × 4–39.

The analysis of the dikaryon *S. commune* 12–43 × 4–39 revealed 142 proteins, 37 of them were solely present with black slate (Supplementary Table S3). They mainly belonged to metabolism and proteins with binding function or cofactor requirement. The 25 proteins exclusively identified without black slate were associated to metabolism, protein fate and protein with binding function or cofactor requirement (Figure 6 and Supplementary Table S4).

Between both life stages, 79 identical proteins were detected. Of these, 12 were exclusively produced in presence of black slate. When considering only predicted secreted proteins, they showed assigned functions in metabolism and cell rescue, defense and virulence. In absence of black slate, ten proteins matched for both strains, and the predicted secreted proteins possessed functions in metabolism or proteins with binding function or cofactor requirement.

From the assigned functions, insights into biological processes during fungal growth with black slate could be gained. To achieve the identification of proteins involved in the process, only proteins exclusively produced during black slate degradation in monokaryon, dikaryon or both were considered. From these, proteins involved in biomass degradation, C-compound metabolism, and stress response were characterized. Many peptidases and glycoside hydrolases (GH) including proteins of GH family 92 and 93, which are generally participating in plant biomass degradation, were secreted. Furthermore, enzymes specifically involved in the degradation of polysaccharides (GH families 5 and 61) or lignin (multicopper oxidases) were secreted. Aside from an intracellular aconitase A/isopropylmalate dehydratase involved in the tricarboxylic acid cycle, proteins associated with carbohydrate metabolism (GH families 5 and 6), polysaccharide metabolism, as well as sugar, glucoside, polyol and carboxylate catabolism (GH family 43, carbohydrate esterase family 4) were detected. Besides these extracellular enzymes, proteome analysis also revealed proteins which are usually intracellularly located and involved in sugar, glucoside, polyol and carboxylate anabolism (phosphoglucose isomerase, aldo/keto reductase, aconitase A/isopropylmalate dehydratase).

Furthermore, proteins involved in stress response (heat shock proteins, peptidyl-prolyl *cis-trans* isomerase) including detoxification processes by export (ABC transporter) and degradation (multicopper oxidase), or thioredoxin, glutaredoxin and glutathione metabolism (gamma-glutamyltranspeptidase) were proven as well as the occurrence of a defense-related protein (osmotin; thaumatin-like protein).

### Differential Regulation of Multicopper Oxidases During Black Slate Utilization

**FIGURE 7:**
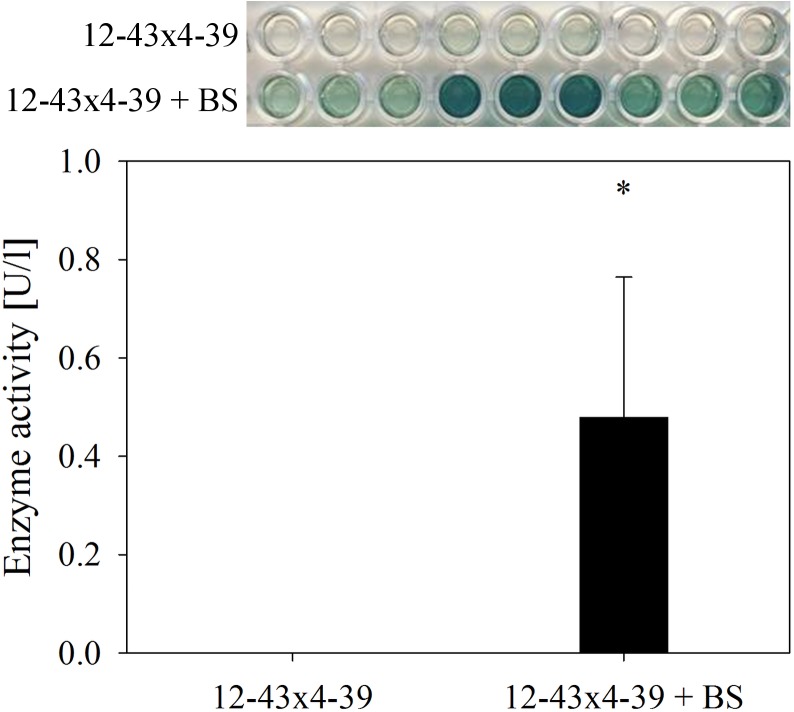
Extracellular laccase activity in *S. commune* 12–43 × 4–39 growing without and with black slate (BS). Error bars represent standard deviation of three biological replicates; ^∗^*p* < 0.05, paired, two-tailed *t*-test.

At the level of enzyme activity, laccases from culture supernatant was measured 7 dpi with an extracellular activity of 0.48 U/l when the dikaryotic strain was grown with black slate (Figure 7). In contrast, no laccase activity was detected in dikaryotic cultures without black slate, or for the monokaryon, neither in absence nor in presence of black slate.

**FIGURE 8:**
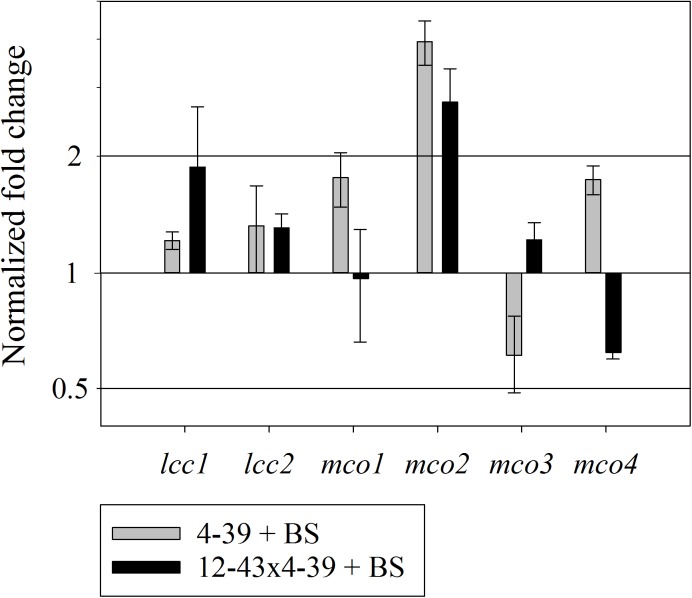
Relative expression of multicopper oxidases in *S. commune* 4–39 and 12–43 × 4–39 during black slate (BS) degradation, normalized by expression for treatments without black slate. Error bars represent standard deviation of three biological replicates.

The expression of all laccase and laccase-like multicopper oxidase genes of *S. commune* was tested for regulation on mRNA level. After growth in half diluted minimal medium with black slate powder, up-regulation of multicopper oxidase *mco2* was found in both life-stages (Figure 8). The other laccase and multicopper oxidase genes did not show changes in expression during treatment with black slate.

## Discussion

### *S. commune* Causes Degradation of Black Slate

The influence of *S. commune* on black slate degradation was tested over a period of 4 weeks. A previous study had suggested the involvement of *S. commune* in release of heavy metals and organic carbon from black slate, when the rock is enzymatically attacked as a substrate (Wengel et al., 2006). Etch pits formed by *S. commune* on the surface of black slate supported the hypothesis of bioweathering (Kirtzel et al., 2017). Here, we could show that both life-stages of the fungus, the haploid monokaryon and the mated dikaryon, caused a decay of rock particles, resulting in a significantly higher turbidity and discoloration of the medium measurable at an absorbance at 450 nm. The dikaryon showed a stronger effect as compared to the monokaryon.

When fungi or bacteria are inoculated with rock samples, an elevated turbidity due to rock compounds like humic substances has been described (Fakoussa, 1988; Hofrichter et al., 1999). Black slates contain high amounts of organic matter which can be, according to their solubility, divided into bitumen or kerogen. They might be rich in humic substances and cause the dark color of the rock. During fungal attack, such organic fractions within the black slate undergo degradation, might dissolve in the medium, and cause its dark colorization. Furthermore, fungal bioweathering might crack solid bitumen or kerogen grains to an average size of 0.7–0.8 μm. Those grains either stick to the hyphae and might be more susceptible to biochemical weathering or remain in the supernatant, are unaffected by centrifugation and thus lead to a further increase in absorbance. Thus, absorbance measurements could be correlated with intensities of fungal-induced black slate degradation.

### Black Slate Influences Fungal Metabolism

During growth of *S. commune* in liquid medium supplemented with black slate, rock grains were attached to the hyphae. Like many organisms, fungi are known to produce extracellular polymeric substances which facilitate the attachment to solid surfaces (Warscheid and Braams, 2000; Gadd, 2007). *S. commune* is a well-known producer of several exopolysaccharides including the glucan schizophyllan (Hao et al., 2010). Therefore, small black slate particles can attach to the mycelial surfaces *via* interaction with the oligo- or polyglucans. Moreover, extracellular polymeric substances frequently contain complex-forming agents and acidic metabolites which in turn can cause an additional accelerated dissolution of black slate (Hoffland et al., 2004; Gorbushina, 2006; Gadd, 2007). Proteome analyses revealed that black slate influenced the tricarboxylic acid cycle where several organic acids like citric, succinic, fumaric and malic acid are produced. They are hypothesized to be deeply involved in rock weathering and could therefore promote black slate degradation (Palmer et al., 1991; Landeweert et al., 2001; Scheerer et al., 2009).

The growth of *S. commune* monokaryon 4–39 was accelerated by black slate. By access to organic materials and released essential nutrients, energy metabolism and biosynthesis are increased allowing for faster growth (Hochella, 2002; Gadd, 2007). However, after 21–28 days, a growth inhibition was scored, likely due to exceeding (heavy) metal concentrations caused by bioweathering of the stone (Gadd, 1993; Baldrian, 2003). Indeed, we could demonstrate that *S. commune* released Pb, U, and exceedingly high concentrations of Al and Fe from black slate. Wengel et al. (2006) have shown an increase in Fe concentrations from 220 μg/l to 330 μg/l and Mn concentrations from 1300 μg/l to 2430 μg/l by different strains of *S. commune* over a period of 84 days. Due to high Al and Fe contents in the black slate used in our study, the release of Fe by *S. commune* is much higher and increases from 140 μg/l to 1180 μg/l within 14 days. Based on these results, *S. commune* might be of interest for industrial bioleaching when addressing the extraction of metals from carbonaceous rocks.

### Wood-Degrading Enzymes Are Responsible for Black Slate Degradation

As a wood-rotting fungus, *S. commune* secretes a broad range of enzymes crucial for degrading complex biomolecules (Ohm et al., 2010). The black slate-specific proteome included proteins involved in C-compound and carbohydrate metabolism, as well as extracellular polysaccharide and lignin degradation. Our results thus link wood-degrading proteins to bioweathering of black slate. In particular, several glycoside hydrolase family proteins and a multicopper oxidase seemed to be involved in degradation of the organic carbon fraction of the rock material. Specifically, *mco2* was up-regulated, which may also be explained by a large number of xenobiotic response elements in the *mco2* promoter (Madhavan et al., 2014). Extracellular enzymes have previously been suggested to participate in fungal rock degradation (Willmann and Fakoussa, 1997; Hofrichter et al., 1999; Silva-Stenico et al., 2007). During the formation of sedimentary organic matter, lignin and lipids were presumed to withstood degradation processes (Durand, 1980) and lignin could be converted into aromatic hydrocarbons (Shen et al., 2016). The organic matter fraction of black slate mainly consists of aromatic hydrocarbons and might thus be susceptible to degradation by ligninolytic enzymes such as laccases (Seifert et al., 2011). Since lignin does not provide a primary source of carbon and energy for fungal growth, it had to be derived from other sources such as cellulose and hemicellulose which provide glucose and other sugars, respectively (Fritsche and Hofrichter, 2005). Black slate organic matter could contain, besides lignin, cellulose and other plant residues (Swanson, 1960), thus explaining the detection of proteins able to degrade plant constituents other than lignin.

### Rock Degradation Causes Stress in *S. commune*

Bioweathering of black slate by *S. commune* was accompanied by a release of metals which might cause stress in fungi. Stress response was visible in significant changes of the proteome, including the multicopper oxidase gene regulation. This enzyme has been shown to be involved in heavy metal stress and could consequently be involved in stress response (Baldrian and Gabriel, 2002; Lorenzo et al., 2006). Since laccases and multicopper oxidases contain four Cu atoms in the active center, the specific sequestration of Cu may be associated with the increased production of laccases/multicopper oxidases. This assumption could also be supported by the metal analysis of the culture medium. Here, the Cu concentrations released from black slate were lower in presence of the fungus. Released Cu was probably accumulated and used for synthesis of the laccase protein.

As a defense mechanism to metal and oxidative stress, fungi are known to produce antioxidants like glutathione or superoxide dismutase (Bellion et al., 2006; Jozefczak et al., 2012). During growth on black slate, the proteome as well as microarray data of *S. commune* showed an up-regulation of antioxidants like thioredoxin, glutaredoxin and glutathione which could protect the fungus from oxidative stress induced by the released (heavy) metals (Carmel-Harel and Storz, 2000; Belozerskaya and Gessler, 2007; Jozefczak et al., 2012; Kirtzel et al., 2017). Furthermore, these extracellular antioxidants could quench the ABTS reaction and prevent a color development during the enzyme assay (Re et al., 1999). This would also explain the low laccase activity in the dikaryon in presence of black slate as well as the inhibition of the ABTS reaction in the monokaryon although proteomics revealed the presence of multicopper oxidase in this strain under black slate treatment.

The complexity and variation in the organic carbon content as well as the presence of high levels of metals in black slate certainly impact bioweathering through *S. commune.* The investigation could show a diverse pattern of secreted proteins and an altered expression for a multicopper oxidase, encoded by the gene *mco2*, which could explain bioweathering and substrate utilization. This proof-of-principle allows a better understanding of the degradation of recalcitrant organic matter through fungal metabolism.

## Author Contributions

JK, KK, and EK conceived and designed the study. SM performed laccase assay and gene expression analyses. JK and YH conducted the protein extraction. NW and AS performed MS analyses and data processing. AB cross-referenced protein data with JGI online databases and JL functionally described the proteins according to the Functional Catalog. JK conducted the rest of the experiments, analyzed and interpreted the data, and wrote the manuscript. EK participated in the critical review of the manuscript.

## Conflict of Interest Statement

The authors declare that the research was conducted in the absence of any commercial or financial relationships that could be construed as a potential conflict of interest.
