# Supplementary material for: Enzymatic Bioweathering and Metal Mobilization From Black Slate by the Basidiomycete Schizophyllum commune
Source: Front Microbiol. 2018 Oct 24;9:2545. doi: 10.3389/fmicb.2018.02545 (PMC6207625; doi:10.3389/fmicb.2018.02545)
Supplement: Supplementary file 1 [file Data_Sheet_1.pdf]

Supplementary Material

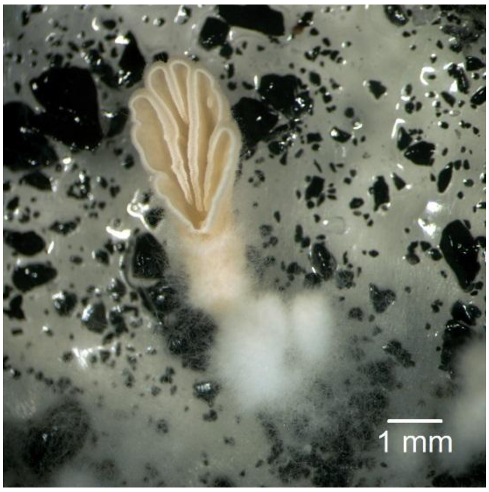

Supplemental Figure S1: Growth and development of *S. commune* dikaryon on water agar with black slate as sole carbon source.

Supplemental Table S1: Regulated proteins of *S. commune* monokaryon 4-39 found in presence of black slate

Protein ID	secreted	JGI Best Hit / Description	FunCat Annotation
2605924	no	Heat shock protein HSS1	Cell rescue, defence and virulence; cellular transport, transport facilitation and transport routes; metabolism; protein fate; protein with binding function or cofactor requirement
2662170	yes	Hypothetical; osmotin, thaumatin-like protein	Cell rescue, defence and virulence
2624233	no	40S ribosomal protein S7 / Ras GTPase, ribosomal protein S7e	Biogenesis of cellular components; cellular communication/signal transduction; cellular transport, transport facilitation and transport routes; metabolism; protein with binding function or cofactor requirement
2604035	yes	Hypothetical protein / Lipase	
2496919	no	Hypothetical; exodeoxyribonuclease	Cell rescue, defence and virulence; cell cycle and DNA processing; metabolism; protein with binding function or cofactor requirement (structural or analytic)
2631822	no	Hypothetical protein / Mitochondrial carrier protein	Cellular transport, transport facilitation and transport routes; protein with binding function or cofactor requirement (structural or analytic); metabolism
2513897	yes	Glycoside hydrolase family 93 protein	
2627185	yes	Hypothetical; peptidase S8/S53	Protein fate; biogenesis of cellular components; metabolism; protein with binding function or cofactor requirement
1164244	no	Hypothetical; DNA/RNA	

		helicase	
2670889	no	Hypothetical	
2516955	yes	Hypothetical; multicopper oxidase	Cell rescue, defence and virulence; metabolism
1039007	no	Hypothetical; zinc finger, C2H2-like	Protein with binding function or cofactor requirement; transcription
2512665	no	Hypothetical; phosphoglucose isomerase	Metabolism; energy
2624086	no	Hypothetical; peptidase M43	Protein fate
2667138	no	Glycoside hydrolase family 43 protein	
1084300	yes	Hypothetical; blue (type 1) copper domain	
2612068	no	Hypothetical; pyruvate kinase	Metabolism; energy; protein with binding function or cofactor requirement
2502504	yes	Glycoside hydrolase family 5 protein	Metabolism; cell rescue, defence and virulence
2573370	no	Hypothetical protein	
2531390	yes	Hypothetical protein	
2630637	no	Hypothetical; aldo/keto reductase	Cell rescue, defence and virulence; energy; interaction with the environment; metabolism; protein with binding function or cofactor requirement
2450004	no	Expressed protein	
2492911	no	Hypothetical protein	
2604628	no	Hypothetical; catalase	Cell cycle and DNA processing; cell fate; interaction with the environment; metabolism; protein with binding function or cofactor requirement; transcription
1184152	yes	Hypothetical; lipase	
2643124	no	Expressed protein	
2645292	yes	Expressed protein	
2565008	yes	Hypothetical; gamma-glutamyltranspeptidase	Metabolism
2635807	no	Hypothetical; peptidyl-prolyl cis-trans isomerase	Systematic interaction with the environment; protein fate; cellular communication/signal transduction mechanism; cell fate; cell rescue, defence and virulence
2643998	no	Hypothetical; aconitase A/isopropylmalate dehydratase	Metabolism; energy; protein with binding function or cofactor requirement
2604063	yes	Hypothetical; peptidase M14	
2722240	no	Hypothetical [Coprinopsis cinerea calmodulin-binding motif protein; Moniliophthora roreri, ID 50%]	

2613271	yes	Glycoside hydrolase family 92 protein	
2200914	no	-	
2522030	no	Hypothetical; CDR ABC transporter	Cell rescue, defence and virulence; cellular transport, transport facilitation and transport routes; metabolism; protein with binding function or cofactor requirement
2639534	yes	Glycoside hydrolase family 6 protein	Metabolism
2603998	yes	Hypothetical protein	
2701674	yes	Hypothetical protein	

Supplemental Table S2: Regulated proteins of *S. commune* monokaryon 4-39 found in absence of black slate

Protein ID	secreted	JGI Best Hit / Description	FunCat Annotation
2547616	yes	Hypothetical; peptidase S10	Protein fate
2670422	yes	Hypothetical; glycoside hydrolase, family 5	Metabolism
2009250	yes	Hypothetical; Peptidase M35	
2611788	no	Translation elongation factor 1a	Protein synthesis; cell fate; protein with binding function or cofactor requirement; regulation of metabolism and protein function
2617616	no	Hypothetical; ATP synthesis coupled proton transport	Cellular transport, transport facilitation and transport routes; energy; interaction with the environment; protein with binding function or cofactor requirement
2621834	yes	Hypothetical protein	Cell rescue, defence and virulence
2684323	no	Hypothetical; protein synthesis factor; translation elongation factor EFG/EF2	Protein synthesis; protein with binding function or cofactor requirement
2604352	yes	Hypothetical; peptidase M35	
2172097	yes	Glycoside hydrolase family 3 protein	Metabolism
2638699	no	Glyceraldehyde-3-phosphate dehydrogenase	
73246	yes	Glycoside hydrolase family 5 protein	Metabolism; cell type differentiation; energy; biogenesis of cellular components; cell fate
2186806	no	Hypothetical; glucose-methanol-choline oxidoreductase	Metabolism; protein with binding function or cofactor requirement; cellular transport, transport facilitation and transport routes
2642124	yes	Hypothetical; alcohol dehydrogenase	Cell rescue, defence and virulence; energy; metabolism
2495926	yes	Glycoside hydrolase	

		family 93 protein	
2611411	no	Hypothetical; thioredoxin	Biogenesis of cellular components; cell cycle and DNA processing; cell rescue, defence and virulence; cellular transport, transport facilitation and transport routes; protein fate
2618843	no	Hypothetical; coproporphyrinogen III oxidase	Metabolism; protein with binding function or cofactor requirement
2498526	no	Tubulin alpha-1B chain	
2009111	yes	Non-catalytic module family EXPN protein	
2606836	no	Hypothetical; phosphoglycerate kinase	Energy; metabolism; protein with binding function or cofactor requirement
1110525	yes	Hypothetical; serine/threonine protein kinase	Metabolism
2625339	no	Hypothetical; phosphoenolpyruvate carboxykinase	Metabolism; energy
2618632	yes	Hypothetical; peptidase S8 and S53	Protein fate; biogenesis of cellular components; metabolism; protein with binding function or cofactor requirement
2597255	yes	Hypothetical; peptidase M36	
2492393	no	Hypothetical; WD40 repeat-containing protein	Cell fate; cell rescue, defence and virulence; cellular communication / signal transduction mechanism; protein fate; protein synthesis; protein with binding function or cofactor requirement; regulation of metabolism and protein function
2642646	no	Hypothetical; GHMP kinase; mevalonate and galactokinase	
237513	yes	Hypothetical; FAD linked oxidase	Metabolism; protein with binding function or cofactor requirement; cellular transport, transport facilitation and transport routes; cell rescue, defence and virulence
2664643	no	Glycoside hydrolase family 16 protein [Coniophora puteana]	
2494852	no	Hypothetical; argininosuccinate synthase	Biogenesis of cellular components; cell cycle and DNA processing; cell fate; cell rescue, defence and virulence; metabolism; protein fate; protein with binding function or cofactor requirement; regulation of metabolism and protein function; transcription
2594763	no	Hypothetical; cytochrome P450	Cellular transport, transport facilitation and transport routes; metabolism
1050459	no	Hypothetical protein	

2636349	no	Hypothetical protein	Metabolism; energy; cell rescue, defence and virulence
2683832	no	Actin-2	
2670385	yes	Hypothetical protein	
2611765	no	40S ribosomal protein S3	Cell cycle and DNA processing; protein synthesis; protein with binding function or cofactor requirement
2515651	yes	Hypothetical; P-loop containing nucleoside triphosphate hydrolases [superfamily]	Biogenesis of cellular components; cell fate; cellular communication / signal transduction mechanism; cellular transport, transport facilitation and transport routes; interaction with the environment; metabolism; protein fate; protein with binding function or cofactor requirement; regulation of metabolism and protein function
2623577	yes	Hypothetical; lipase	Metabolism; cell rescue, defence and virulence; interaction with the environment
1161886	no	Hypothetical; ankyrin	Biogenesis of cellular components; cellular communication / signal transduction mechanism; cellular transport, transport facilitation and transport routes; metabolism; protein fate; protein with binding function or cofactor requirement; regulation of metabolism and protein function
2640541	no	Hypothetical; [Verticillium dahliae endoribonuclease L-PSP]	
2610771	no	Hypothetical; glucose-methanol-choline oxidoreductase	Metabolism; protein with binding function or cofactor requirement; cellular transport, transport facilitation and transport routes
2520972	yes	Hypothetical; peptidase S10	Protein fate
2617541	no	FK506-binding protein; peptidyl-prolyl cis-trans isomerase	Cell cycle and DNA processing; cell rescue, defence and virulence; cellular communication / signal transduction mechanism; interaction with the environment; protein fate; protein synthesis; protein with binding function or cofactor requirement; regulation of metabolism and protein function
2597567	yes	Expressed protein	
1176082	yes	Hypothetical; peptidase M28, calcium-binding EF-hand	Protein fate
2607034	yes	Glycoside hydrolase family 13 protein	Metabolism
2618543	no	Mitochondrial carrier protein	Cellular transport, transport facilitation and transport routes; interaction with the environment

2625433	yes	Glycoside hydrolase family 61 protein	Metabolism
2698782	no	Hypothetical; DNA repair/transcription protein Mms19	Biogenesis of cellular components; cell cycle and DNA processing; cell rescue, defence and virulence; metabolism; transcription
2631957	no	Hypothetical; peptidase M24, creatinase	Protein fate; protein with binding function or cofactor requirement
1050936	no	Hypothetical; [Serpula lacrymans]	
2615411	no	Hypothetical; UspA	Cell rescue, defence and virulence
2490682	no	Hypothetical protein	
2642638	no	Hypothetical protein	
2644822	no	Hypothetical; Porin	Biogenesis of cellular components; cell fate; cellular transport, transport facilitation and transport routes; energy; interaction with the environment; protein with binding function or cofactor requirement
2086990	yes	Hypothetical protein	
2643673	no	Hypothetical; putative N2,N2-dimethylguanosine tRNA methyltransferase	Biogenesis of cellular components; protein with binding function or cofactor requirement; protein fate
2326475	yes	Hypothetical protein	
2622559	yes	Hypothetical; peptidase M36	
2612243	no	Hypothetical; glutamine amidotransferase class-I	Metabolism
2631700	yes	Hypothetical; fungal specific transcription factor	Transcription
2628084	yes	Hypothetical; peptidase M35	Protein fate; protein with binding function or cofactor requirement
1115145	no	Hypothetical protein	
1133652	no	Hypothetical; post-SET zinc-binding region	Biogenesis of cellular components; cell cycle and DNA processing; cell type differentiation; cellular communication / signal transduction mechanism; metabolism; protein fate; protein with binding function or cofactor requirement; regulation of metabolism and protein function; transcription
2616532	no	Hypothetical; adenosine/AMP deaminase	Energy; metabolism
1169398	no	Hypothetical; serine/threonine protein kinase	Cell fate; cell rescue, defence and virulence; cellular communication / signal transduction mechanism; metabolism; protein fate; protein with binding function or cofactor requirement; regulation of metabolism and protein function
2665914	no	Hypothetical protein	

55993	yes	Hypothetical; peptidase M35	
2662991	yes	Hypothetical; peptidase M35	Protein fate; protein with binding function or cofactor requirement
2606470	yes	Hypothetical; C-type lectin	
2635003	no	Glycoside hydrolase family 18 protein	Metabolism; biogenesis of cellular components
2627742	yes	Hypothetical; chitin binding [Moniliophthora roreri, 59%]	

Supplemental Table S3: Regulated proteins of *S. commune* dikaryon 12-43x4-39 found in presence of black slate

Protein ID	secreted	JGI Best Hit / Description	FunCat Annotation
2100685	yes	Hypothetical; chitinase; carbohydrate-binding module family 50 protein	
2489112	yes	Hypothetical; thaumatin, osmotin, thaumatin-like protein [superfamily]	
2009111	yes	Non-catalytic module family EXPN protein	
2492393	no	Hypothetical; WD40 repeat-containing protein	Cell fate; cell rescue, defence and virulence; cellular communication / signal transduction mechanism; protein fate; protein synthesis; protein with binding function or cofactor requirement; regulation of metabolism and protein function
2664643	no	Glycoside hydrolase family 16 protein [Coniophora puteana]	
2620247	yes	Hypothetical; E3 ubiquitin ligase	
2662170	yes	Hypothetical; osmotin, thaumatin-like protein [superfamily]	Cell rescue, defence and virulence
2618997	yes	Carbohydrate esterase family 4 protein	Metabolism; cell type differentiation
2604035	yes	Hypothetical; lipase	
2619343	yes	Hypothetical; peptidase A1	Cell fate; cell rescue, defence and virulence; cell type differentiation; interaction with the environment; protein fate; protein with binding function or cofactor requirement; transposable elements, viral and plasmid routes
2670889	no	Hypothetical protein	
2617902	no	Hypothetical; lysine acetyltransferase	

		[Termitomyces sp. 40%]	
2086990	yes	Hypothetical protein	
2615368	yes	Hypothetical; endonuclease/ exonuclease/phosphatase, DNase I-like [superfamily]	
2667138	no	Glycoside hydrolase family 43 protein	
2502504	yes	Glycoside hydrolase family 5 protein	Metabolism; cell rescue, defence and virulence
2573370	no	Hypothetical protein	
2604628	no	Hypothetical; catalase	Cell cycle and DNA processing; cell fate; interaction with the environment; metabolism; protein with binding function or cofactor requirement; transcription
1176926	no	Hypothetical; peptidase S41	
2643124	no	Expressed protein	
2645292	yes	Expressed protein	
2239711	yes	Expressed protein	
2611919	yes	Hypothetical; aldehyde dehydrogenase	
2565008	yes	Hypothetical; gamma-glutamyltranspeptidase	Metabolism
2611714	no	predicted protein [Laccaria bicolor]	
2612695	yes	Hypothetical; lipase	
2665914	no	Hypothetical protein	
2606470	yes	Hypothetical; C-type lectin	
2722240	no	Hypothetical; [Coprinopsis cinerea calmodulin-binding motif protein; Moniliophthora roreri, 50%]	
2613271	yes	Glycoside hydrolase family 92 protein	
2516634	yes	Hypothetical; heterokaryon incompatibility protein Het-C; collagens (type IV and type XIII)	Cell type differentiation
2628203	yes	Glycoside hydrolase family 61 protein	Metabolism
2581649	no	Hypothetical; DNA/RNA helicase	Cell cycle and DNA processing; protein with binding function or cofactor requirement
2640978	no	Hypothetical; carbohydrate esterase family 16	

2627223	yes	Hypothetical protein	
2605157	yes	Glycoside hydrolase family 43 protein	Metabolism
2608082	yes	Hypothetical; mitochondrial import inner membrane translocase subunit [Coprinopsis cinerea 53%]	Cellular transport, transport facilitation and transport routes; protein fate

Supplemental Table S4: Regulated proteins of *S. commune* dikaryon 12-43x4-39 found in absence of black slate

Protein ID	secreted	JGI Best Hit / Description	FunCat Annotation
2621834	yes	Hypothetical protein	Cell rescue, defence and virulence
2612719	yes	Glycoside hydrolase family 11	
2641020	yes	Carbohydrate esterase family 4	Metabolism; protein with binding function or cofactor requirement
2604352	yes	Hypothetical; peptidase M35	
2172097	yes	Glycoside hydrolase family 3	Metabolism
2642372	no	Hypothetical; delta-endotoxin CytB	
2492871	yes	Glycoside hydrolase family 16	Metabolism
2615052	yes	Glycoside hydrolase family 79	
2712269	yes	Hypothetical protein	
2515651	yes	Hypothetical; P-loop containing nucleoside triphosphate hydrolases [superfamily]	Biogenesis of cellular components; cell fate; cellular communication / signal transduction mechanism; cellular transport, transport facilitation and transport routes; interaction with the environment; metabolism; protein fate; protein with binding function or cofactor requirement; regulation of metabolism and protein function
2502024	yes	Hypothetical; glycosyl hydrolases family 32	Metabolism
2631822	no	Hypothetical; mitochondrial carrier protein	Cellular transport, transport facilitation and transport routes; protein with binding function or cofactor requirement Metabolism
2627185	yes	Hypothetical; peptidase S8 and S53	Protein fate; biogenesis of cellular components; metabolism; protein with binding function or cofactor requirement
2499350	yes	Hypothetical; Peptidase	Protein fate; biogenesis of cellular

		S8 and S53	components; metabolism; protein with binding function or cofactor requirement
2612243	no	Hypothetical; glutamine amidotransferase class-I	Metabolism
2553746	no	Predicted protein [Postia placenta]	
2631700	yes	Hypothetical; fungal specific transcription factor	Transcription
2731636	no	Hypothetical; GTP-binding protein, HSR1-related	Cell cycle and DNA processing; cellular transport, transport facilitation and transport routes; protein synthesis; protein with binding function or cofactor requirement; transcription
2628084	yes	Hypothetical; peptidase M35	Protein fate; protein with binding function or cofactor requirement
1133652	no	Hypothetical; post-SET zinc-binding region	Biogenesis of cellular components; cell cycle and DNA processing; cell type differentiation; cellular communication / signal transduction mechanism; metabolism; protein fate; protein with binding function or cofactor requirement; regulation of metabolism and protein function; transcription
2616532	no	Hypothetical; adenosine/AMP deaminase	Energy; metabolism
2634024	yes	Hypothetical; peptidase A1	Cell fate; cell rescue, defence and virulence; cell type differentiation; interaction with the environment; protein fate; protein with binding function or cofactor requirement; transposable elements, viral and plasmid routes
2632434	no	Hypothetical; fungal specific transcription factor	Metabolism; protein with binding function or cofactor requirement; transcription
1169398	no	Hypothetical; serine/threonine protein kinase	Cell fate; cell rescue, defence and virulence; cellular communication / signal transduction mechanism; metabolism; protein fate; protein with binding function or cofactor requirement; regulation of metabolism and protein function
2604063	yes	Hypothetical; peptidase M14	
